# An iterative noisy annotation correction model for robust plant disease detection

**DOI:** 10.3389/fpls.2023.1238722

**Published:** 2023-10-13

**Authors:** Jiuqing Dong, Alvaro Fuentes, Sook Yoon, Hyongsuk Kim, Dong Sun Park

**Affiliations:** ^1^ Department of Electronic Engineering, Jeonbuk National University, Jeonju, Republic of Korea; ^2^ Core Research Institute of Intelligent Robots, Jeonbuk National University, Jeonju, Republic of Korea; ^3^ Department of Computer Engineering, Mokpo National University, Muan, Republic of Korea

**Keywords:** annotation correction, plant disease detection, auto-labeling, noisy labels, teacher-student model

## Abstract

Previous work on plant disease detection demonstrated that object detectors generally suffer from degraded training data, and annotations with noise may cause the training task to fail. Well-annotated datasets are therefore crucial to build a robust detector. However, a good label set generally requires much expert knowledge and meticulous work, which is expensive and time-consuming. This paper aims to learn robust feature representations with inaccurate bounding boxes, thereby reducing the model requirements for annotation quality. Specifically, we analyze the distribution of noisy annotations in the real world. A teacher-student learning paradigm is proposed to correct inaccurate bounding boxes. The teacher model is used to rectify the degraded bounding boxes, and the student model extracts more robust feature representations from the corrected bounding boxes. Furthermore, the method can be easily generalized to semi-supervised learning paradigms and auto-labeling techniques. Experimental results show that applying our method to the Faster-RCNN detector achieves a 26% performance improvement on the noisy dataset. Besides, our method achieves approximately 75% of the performance of a fully supervised object detector when 1% of the labels are available. Overall, this work provides a robust solution to real-world location noise. It alleviates the challenges posed by noisy data to precision agriculture, optimizes data labeling technology, and encourages practitioners to further investigate plant disease detection and intelligent agriculture at a lower cost. The code will be released at https://github.com/JiuqingDong/TS_OAMIL-for-Plant-disease-detection.

## 1 Introduction

According to the United Nations, the world population reached 8 billion in mid-November 2022 (Pison, 2022). Meanwhile, hunger-related fatalities rose and reached 4 million in 2020, 10 times the number of COVID-19 fatalities in the same period (He and Krainer, 2020). Given those antecedents, it is essential to find ways to feed a growing population while limiting environmental damage and improving the yield and quality of agricultural products (Smith et al., 2022). Nonetheless, this endeavor presents a formidable challenge given the susceptibility of crops to afflictions and stressors, both of which have the potential to engender detrimental economic repercussions and reductions in production output. Consequently, the timely identification of preliminary indications of disease and stress factors in vegetation assumes paramount significance in instituting optimal conditions conducive to crop cultivation.

Recent frontiers in non-invasive sensor technology and image processing methodologies provide potential remedies for the aforementioned challenges. Deep learning methods have shown great success in various tasks, such as plant state monitoring (Xu et al., 2021a; Bhise et al., 2022; Wang et al., 2022a; Dong et al., 2023; Shoaib et al., 2023; Tomaszewski et al., 2023), medical diagnosis (Yao et al., 2022; Nalepa et al., 2023), cell variation (Rahman et al., 2021), and flora (Evangelisti et al., 2021; Ganesh et al., 2022). These achievements frequently hinge upon the extraction of visual cues from images and the provision of precise annotations. Nevertheless, annotating these domain-specific datasets is not as simple as identifying cats and dogs. Acquiring an accurately annotated dataset relies on expert knowledge, which is only sometimes feasible. Deploying current deep learning-based methods in real-world applications may suffer primarily from limited and imperfect data (Xu et al., 2023). In a real scenario, practitioners without computer vision knowledge lack experience in annotating high-quality boxes, and annotators without domain knowledge have difficulties in annotating accurate object boxes. Annotation cost would be significantly high if domain experts were to annotate the entire dataset. Embracing these imperfect annotations is a promising strategy that has not received sufficient attention (Dubel et al., 2023). We need to consider the practical problem of whether it is worth spending more on the expert and computational costs to get a better performance or applying techniques to mitigate these issues.

We attempt to answer this question in the general case. As illustrated in previous work (Dong et al., 2022), enhancing performance through increased computational expenditure is prevalent in computer vision techniques. For instance, as observed in (Liu et al., 2022c), the image classification accuracy on ImageNet only improved by 6%, while the number of parameters increased from 88 million to 30 billion. Compared to computational cost, labor cost for annotation is more substantial. Particularly, researchers refine the label set multiple times just for a weak improvement in a specific task. Therefore, refinement labeling is high-cost and low-reward, while spending a considerable cost for a slight improvement in practical applications is unwise.

Compared to class noise, fully supervised object detector (FSOD) performance is more susceptible to inaccurate localization (Dong et al., 2022). Note that FSOD means that each instance is assigned an accurate label during the training phase. Moreover, localization noise is almost unavoidable compared to class noise, because not all researchers can afford such colossal labor or time costs and organize a professional processing line for annotation as the COCO team (Lin et al., 2014) has done. As a result, researchers are often faced with dealing with a noisy label set. In this study, we address these challenges by considering a method capable of handling noisy annotations to mitigate their impact. This allows us to relax the strict annotation standards, which in turn benefits intelligent agricultural practitioners by reducing the threshold for their involvement.

**Figure 1:**
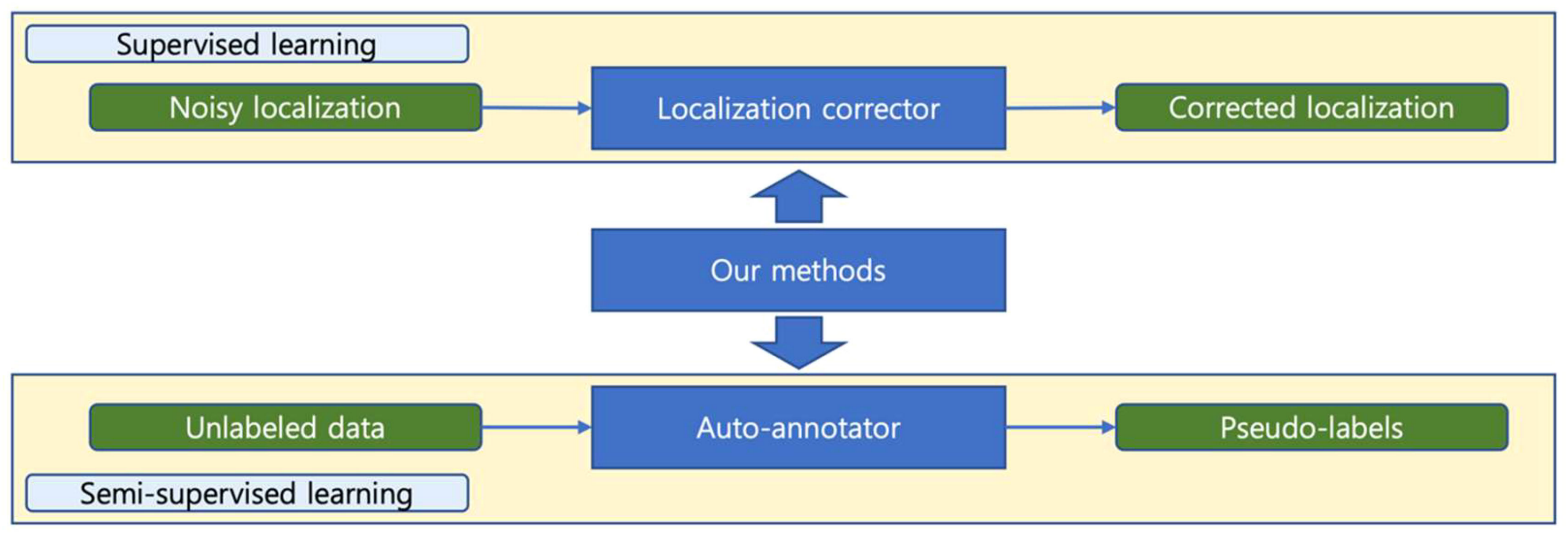
Two application scenarios of our method. In supervised learning, our method can correct noisy locations, while in semi-supervised learning, it can generate accurate pseudo-labels from unlabeled data. Our method plays different roles in different settings.

Based on the above-mentioned, in this paper, we propose an annotation correction strategy based on the teacher-student learning paradigm, which is effective in two training settings. Our method improves the model’s performance in supervised learning tasks by correcting for noisy localization noise. While in semi-supervised learning tasks, it can act as an automatic annotator, generating accurate pseudo-labels. **Figure 1** demonstrates two main application scenarios of our method.

Regarding the correction of class noise, there are already a few methods for training accurate Deep Neural Networks (DNNs) under noisy labels (Li et al., 2020; Mao et al., 2021; Xu et al., 2021c; Song et al., 2022; Huang et al., 2023; Zhang et al., 2023). A significant line of research focuses on the classification task, which develops various techniques to deal with noisy labels, such as sample selection (Xia et al., 2021), robust regularization (Gudovskiy et al., 2021; Huang et al., 2023), and robust loss functions (Jiang et al., 2021). In contrast, there are significantly fewer studies focused on addressing localization noise. Recently, some efforts have extended the accumulated experience in classification to object detection tasks, such as class noise correction (Xu et al., 2021c), missing label correction (Xu et al., 2019), and noisy localization correction (Liu et al., 2022a). However, these methods mainly focus on general vision datasets, such as MS-COCO (Lin et al., 2014), PASCAL VOC (Everingham et al., 2010), and ImageNet (Deng et al., 2009), rather than domain-specific datasets. Although, there are a large number of frontier works in plant disease detection (Fuentes et al., 2018; Nazki et al., 2020; Fuentes et al., 2021; Xu et al., 2021b; Khakimov et al., 2022; Priyadharshini and Dolly, 2023), which can achieve 90% or even higher performance on their respective datasets, these methods assume in advance that they are trained on well-annotated datasets. Unlike previous approaches, we emphasize that our model is trained on noisy annotations.

Our paper further studies the distribution of localization noise and the noise synthesis rules. Intuitively, the localization of a small object noise seems more severe than large objects. Based on this motivation, this paper provides an insightful analysis of the distribution of location noise and the relationship between noise distribution and bounding box size. Previous research work generally synthesized noise by perturbing clean bounding boxes (Liu et al., 2022a), while synthesized noise follows a uniform distribution $relative_{noise}∼U\left(-r,\;r\right)$ . The r is a parameter to control the noise level. We argue that such a noise synthesis rule without considering object size is unreasonable. Unlike (Liu et al., 2022a), our method is trained on a dataset with real-world noise and synthesized noise following the real-world distribution.

**Figure 2:**
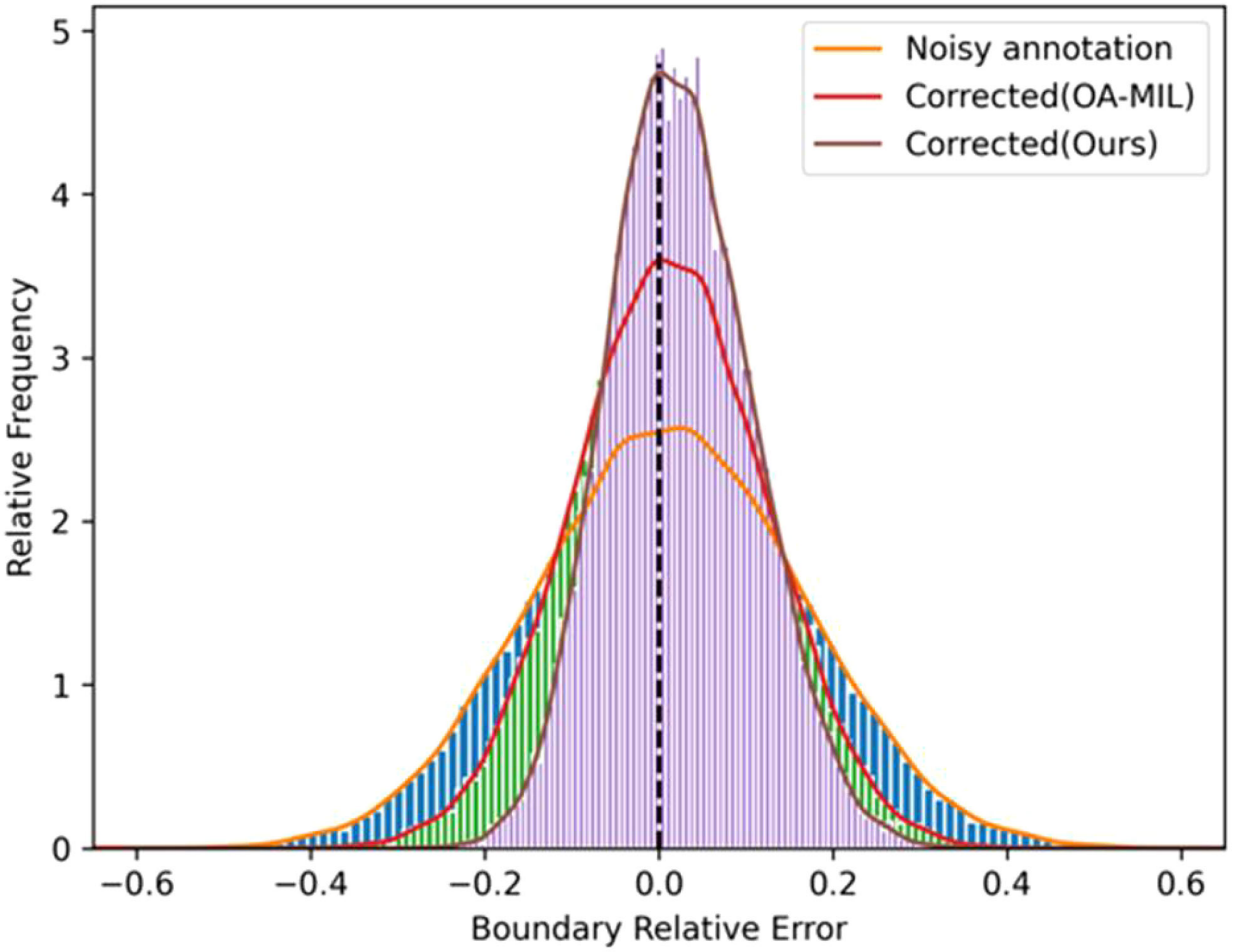
Distributions of relative boundary coordinate errors for noisy annotations and our corrected ones.

To build a robust detector, we expect the model learns from corrected labels rather than noisy labels. Therefore, we introduce an iterative teacher-student learning framework on the correction network (Liu et al., 2022a). Teacher-student learning is a learning paradigm, introduced in knowledge distillation (Gou et al., 2021), where knowledge is usually distilled from a teacher network to improve the feature representation of students (Wang et al., 2022b). Typically, a teacher model is more complicated than a student, but a simple student network can achieve comparable performance to a teacher. Unlike most existing teacher-student algorithms (Hu et al., 2022), teacher and student networks, in this work, hold the same architecture but different parameters. We train a teacher network on noisy datasets and the corrected annotations are used as a supervised signal for the student network. In addition, we found that labels are still noisy after being corrected by OA-MIL (Liu et al., 2022a) (refer to the red line in **Figure 2**). Therefore, a teacher-student learning paradigm is adopted to correct noisy labels iteratively. In other words, the performance improvement of the student model comes from the optimized label set, which can be regarded as the additional knowledge of the teacher model. Our method avoids manually refining noisy labels, thereby reducing annotation costs.

To further reduce annotation costs, semi-supervised and unsupervised learning algorithms are usually used to learn the feature representation from unlabeled data. However, unsupervised learning is often used to tackle more complex tasks such as domain adaptation (Liu et al., 2022b) and learning from compression (He et al., 2022), which requires a large-scale dataset. In addition, unsupervised learning cannot provide an intuitive category label like semi-supervised learning, which is not friendly to plant disease detection. Therefore, semi-supervised learning is more suitable for handling general plant disease detection tasks. Semi-supervised object detection tasks aim to train an object detector with many image-level annotations and a few box-level annotations (Li et al., 2022) and generate many instance-level pseudo-labels. Suppose the pseudo-labels generated during the semi-supervised learning process are regarded as a collection of noisy labels. Our method can naturally extend to semi-supervised learning tasks, requiring only limited box-level annotations and no extra image-level annotations. To the best of our knowledge, this is the first semi-supervised learning method in plant disease detection, even though it is not explicitly designed for the semi-supervised learning task.

Overall, our primary contributions can be summarized as follows:

1. We investigate the distribution of location noise in real-world plant data annotation and provide insightful analyses.
2. An annotation correction network based on the iterative teacher-student learning paradigm is proposed to offset the impact of noise on model performance by correcting imprecise labeled boxes.
3. Our method can be easily extended to semi-supervised learning tasks and automatic labeling. For instance, we achieve approximately 75% of the performance of fully supervised learning methods using only 1% of accurate labels.
4. Our approach lowers the labeling quality requirements for practitioners, by improving the robustness of the model to localization noise. Additionally, it is anticipated to advance applications related to location-based tasks.

The remaining sections of the paper are organized as follows. Section 2 introduces the dataset used for noise analysis, the results of the noise analysis, and the correction methods. Section 3 presents the experimental results, including the correction results, experimental results in the semi-supervised setting, and ablation experiments. Section 4 discusses relevant topics, limitations, and future work. Section 5 provides a conclusion for the paper. We have included all qualitative results of the experiments in the appendix, denoted as **Figure A** in the main text, where A represents the appendix.

## 2 Materials and methods

### 2.1 Datasets

The paprika disease dataset (Dong et al., 2022) was used to evaluate our methods. As previously noted, researchers often engage in iterative model optimization and label set refinement throughout the task processing to enhance the ultimate performance. Indeed, the dataset in (Dong et al., 2022) was refined through multiple mutual verifications between plant experts and artificial intelligence experts to achieve a well-annotated dataset finally. Therefore, based on the assumption that the paprika disease dataset is clean, we conducted experiments and analyses. Please note that the unrefined raw label set is called the real-world noisy dataset, and the well-annotated label set is called the clean dataset. Both followed the split strategy of the dataset in (Dong et al., 2022). The Paprika disease dataset consists of five disease categories, with 5,928 images.

If the pseudo-labels generated during the semi-supervised learning process are considered as noisy labels, our method can be employed to correct location noise. However, besides positional noise, pseudo-labels also come with class noise. Therefore, it is essential to evaluate the performance of positional correction using a single-class object detection dataset to avoid the influence of multi-class classification problems on our model. Unlike the paprika disease dataset, the Global Wheat Head Detection (GWHD2021) (David et al., 2020; David et al., 2021) dataset only requires the distinction of wheat heads and does not involve multi-class classification. Thus, we evaluated our method in a semi-supervised setting using GWHD2021. Please note that GWHD2021 is only used for evaluating the performance of our method when extended to the semi-supervised setting. While handling the labels, we found that some images had no annotation information, totaling 128. Therefore, we used a total of 6,387 images that had annotations. We followed their dataset split scheme. More details about the dataset are presented in **Table 1**.

**Table 1:** Details of datasets and splits.

(A)Paprika dataset (Dong et al., 2022)				
Category	Training	Validation	Test	Total
Blossom end rot	933	117	133	1183
Gray mold	355	43	43	441
Powdery mildew	334	37	45	416
Spider mite	347	33	40	420
Spotting disease	2773	367	328	3468
(B)GWHD2021 (David et al., 2020; David et al., 2021)				
Category	Train	Validation	Test	Total
Wheat Head	3605	1448	1334	6387

### 2.2 Preliminaries on object detection

Most object detectors follow the dense prediction paradigm, usually with an ingenious loss function to guide them to predict correctly. Generally, a loss function in object detection mainly consists of two parts: classification loss and regression loss. Classification loss is used to distinguish categories, while regression loss is designed to localize objects, which can be abstracted as Equation 1:


(1) $$\begin{array}{c} L\left({\hat{C}}_{i},{\hat{B}}_{i}\right)=\lambda_{cls}\sum_{i}L_{cls}\left({\hat{C}}_{i},\;C_{i}\right)+\lambda_{reg}\sum_{i}I_{\left\{C_{i}\neq 0\right\}}L_{reg}\left({\hat{B}}_{i},\;B_{i}\right) \end{array}$$

where λ denotes the normalization and reweighting factor. ${\text{L}}_{\text{c}ls}$ and ${\text{L}}_{reg}$ are classification loss and regression loss, ${\hat{C}}_{i}$ and ${\hat{B}}_{i}\;$ denote the prediction results of category and location, $C_{i}$ and $B_{i}$ denote the ground truth of category and location. $I_{\{C_{i}\neq 0\}\;}$ is the binary function, being one if $C_{i}\neq 0$ (foreground) and zero otherwise (background).

In fully supervised object detection, the default for evaluating the dataset is treated as accurate ground truth labels without considering possible human errors, which means that the regression object $B_{i}$ in Equation 1 is considered accurate. Due to the high cost to elaborate annotation, some publicly available datasets may satisfy the above criteria. However, some private and self-collected datasets usually do not meet these conditions. To explore the impact of noise in real-world scenarios, we studied the form of the distribution of the noisy label set.

### 2.3 Location noise analysis

To quickly obtain a label set with real-world noise, we used the raw label set that has yet to be refined. Furthermore, our real-world noisy dataset shares almost the same class noise and missing labels as the clean ones to ensure a fair comparison. The only difference between noisy and clean label sets is that the location of bounding boxes in the noisy label set is more inaccurate. Therefore, we analyze real-world localization noise by traversing and matching boxes of the same category in two different versions of the annotation files. Specifically, in the clean and noisy label pairs, we compute the intersection between each clean bounding box and all noisy bounding boxes under the same category, selecting the largest intersection as the matching box. Then, we calculate the difference between this box and its corresponding matching box. For analysis, the absolute error is defined as the difference between each boundary of the clean bounding box and the corresponding boundary of its noise bounding box. The relative error is defined as the ratio of the absolute to the width or height of the clean bounding box. We analyzed the location noise distribution from the noisy label set, leading to three interesting observations: 1. The absolute error is proportional to the corresponding bounding box width or height; 2. The relative error related to the bounding box width and height follows a Gaussian distribution; 3. The mean of relative error is smaller than 0 in objects with a large width or height, while it is greater than 0 with a small width or height.

**Figure 3:**
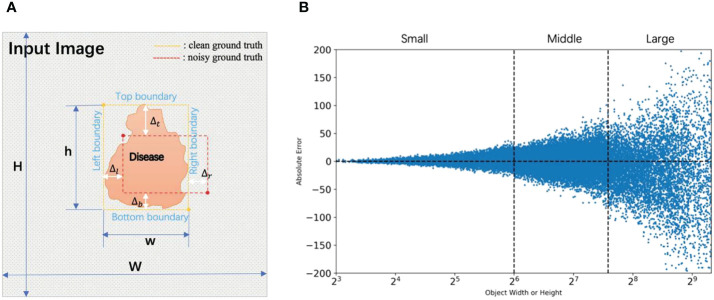
The definition and distribution of the boundary noise. **(A)** An example of the noise of four boundaries. Four white gaps correspond to four boundary noises (Δ_*_). **(B)** Scatter diagram of absolute error for each boundary coordinate with respect to corresponding bounding box width or height. Width and height are scaled to 640 x 640.

**Figure 3A** defines four boundaries and absolute error in a bounding box, where (H, W) denotes image width and height, and (h, w) denotes clean bounding box width and height. For small, middle, and large bounding box sizes, we refer to the definition of the COCO dataset (Lin et al., 2014). For example, h is defined as small if h/H∈(0, 0.1] , while h is defined as large if h/H∈(0.3, 1] . **Figure 3B** shows the scatter plot of the relationship between the absolute error of four boundaries and the bounding box width or height.

**Figure 4:**
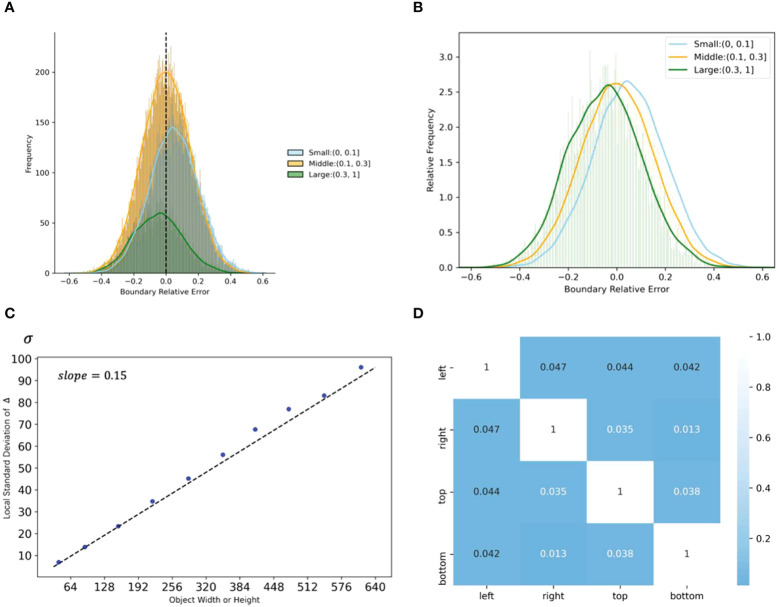
Analyses of location noise in the real-world noisy dataset. **(A)** The distribution of relative errors for different sizes of width and height. **(B)** Relative frequency of relative errors by size. **(C)** The standard deviation by sizes. **(D)** Correlation coefficients between relative errors of different boundaries. Please note that width and height are normalized.


**Figures 4** shows the relative error distribution, the mean of absolute error by size, and correlation analysis for four types of boundary noises. **Figure 4A, B** indicate that boundary noises follow the normal distribution. We introduce the root mean square relative error γ to measure the noise level. By computing the location noise for all boundaries, we get γ=0.15 in a real-world noisy label set. The root mean square relative error is calculated by Equation 2:

(2) $$\begin{array}{c} \gamma =\sqrt{\frac{1}{n}\sum_{i=1}^{n}{\left(\frac{{\text{Δ}}_{i}}{S_{i}}\right)}^{2}} \end{array}$$

where ${\text{Δ}}_{i}$ represents the absolute error of the noise and $S_{i}$ represents the width or height of the bounding box. Assuming that the absolute error standard deviation is σ , then σ can be expressed by Equation 3:


(3) $$\begin{array}{c} \sigma =\sqrt{\frac{1}{n}\sum_{i=1}^{n}{\left({\text{Δ}}_{i}\right)}^{2}} \end{array}$$

From (2) and (3), the relationship between σ and γ can be obtained by Equation 4:


(4) $$\begin{array}{c} \sigma =\sqrt{\frac{1}{n}\sum_{i=1}^{n}[{\left(\frac{{\text{Δ}}_{i}}{S_{i}}\right)}^{2}\;\cdot \;S_{i}^{2}]}\approx \gamma \cdot {\sqrt{E\left(S_{i}\right)}}^{2} \end{array}$$

**Figure 4C** shows the relationship between σ in Equation3 and bounding box size, where each point represents the local standard deviation within a specific range. For example, the abscissa of the first point represents the mean value of the bounding box size between 0 and 64, and the ordinate represents the σ in Equation3 of the corresponding range. The slope of the fitted line is approximately the same as γ , as expressed in Equation 4. Besides, **Figure 4D** shows that the noise distribution of the four boundaries is weakly correlated, thus, γ can share on the four boundaries to synthesize the noise.

**Figure 5:**
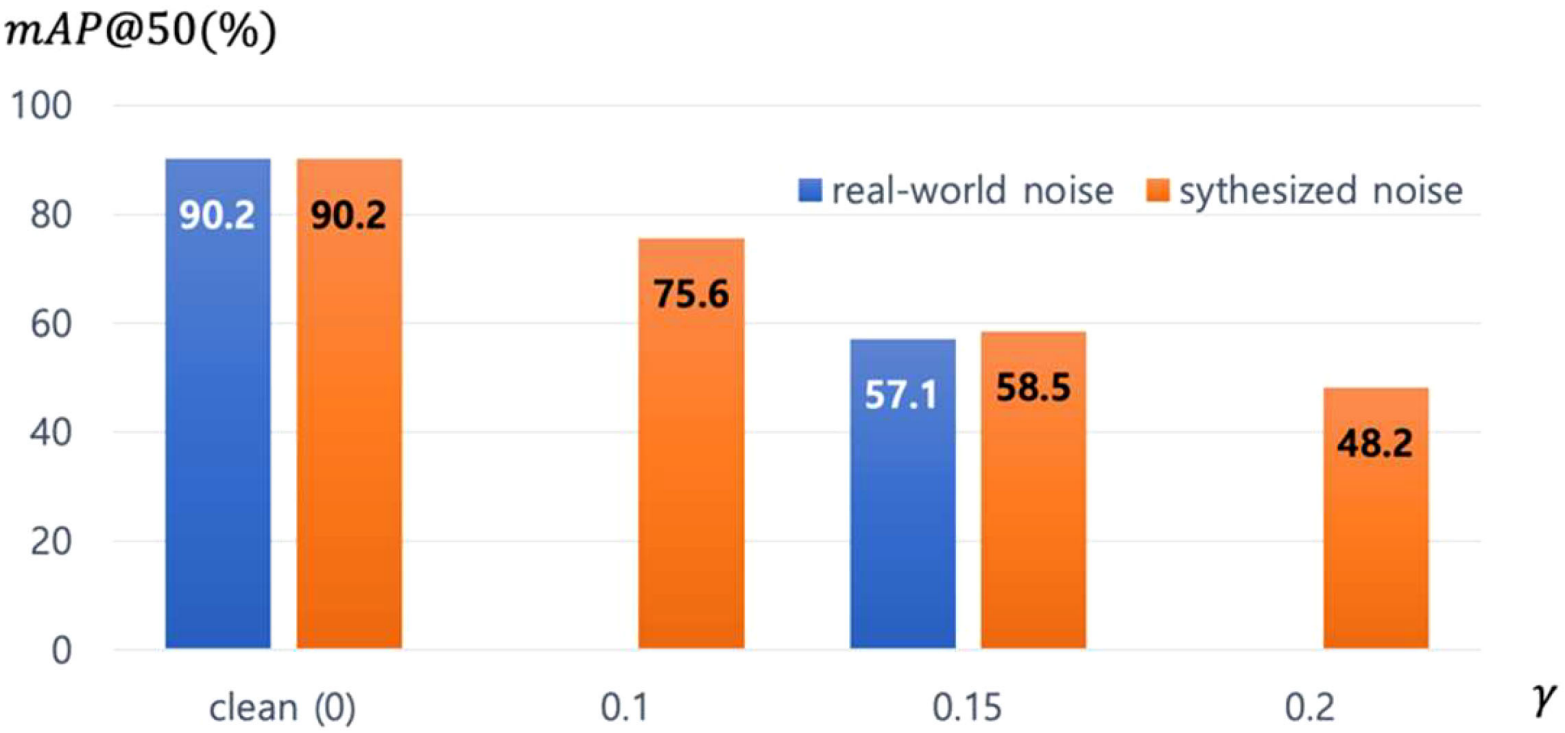
The impact of real-world noise versus synthesized noise on Faster-RCNN. The *γ* equals 0.15 in real-world noise.

Overall, the relative error distribution follows a normal distribution with mean 0. To obtain the synthetic noise label set, we add absolute error ${\text{Δ}}_{i}$ to the four boundaries of each bounding box, ${\text{Δ}}_{i}∼N\left(0,{\left(\gamma \cdot S_{i}\right)}^{2}\right)$ . We generated synthetic noise datasets by changing γ and used them to train Faster-RCNN (Ren et al., 2015) detector. **Figure 5** shows the performance according to the datasets used in the training. We can see that the performance of the detector trained with a real noisy dataset with γ=0.15 and the detector trained with a synthetic dataset made with γ=0.15 are relatively similar, indicating that our noise synthesis method and analysis are reasonable and realistic.

### 2.4 Robust detector via iterative noisy annotation correction model

Researchers often face a dataset with noisy location annotations due to limited labor or time budgets. Therefore, it is worth exploring how to utilize these noisy annotations effectively, as using them directly can lead to notable performance degradation. Liu et al. (2022a) propose an object-aware multi-instance learning (OA-MIL) approach that jointly optimizes an instance selector, an instance classifier, and an instance generator. They tried to generate high-quality proposals guided by noisy annotations. Specifically, in OA-MIL, an instance generator is used to generate multiple proposal boxes near each noisy annotated bounding box. These proposal boxes are generated to capture potential instances of the target object. Subsequently, an instance classifier is employed to assess the probability of containing the target object within these proposal boxes. The instance classifier predicts whether the proposal boxes contain the desired target. Then, the instances selector considered the proposals and the noisy ground truth to select the corrected bounding box. The corrected bounding box was used as a supervised signal to update network parameters. Finally, OA-MIL merges proposal boxes and the noisy ground truth, which is formulated as follows:


(5) $$\begin{array}{c} {\;}_{\text{Bi}}^{\text{correct}}=\lambda \cdot {\;}_{\text{Bi}}^{*}+\left(1-\lambda \right)\cdot {\;}_{\text{Bi}} \end{array}$$

where ${\;}_{\text{Bi}}^{\text{correct}}$ denotes the corrected bounding box. ${\;}_{\text{Bi}}^{*}$ and ${\;}_{\text{Bi}}$ denote the best proposal and noisy bounding box, respectively. λ denotes the normalized weight factor.

Inaccurate ground truth dominates strong object localization priors. In some cases, poor instance initialization could render failure during training, resulting in no performance gains or even worse. Although the corrected bounding boxes have lower γ than the noisy annotations, only relying on the annotations corrected to provide appropriate supervision still cannot achieve the optimal performance of the model.

To address the above issues, we employed a teacher-student learning framework in the training process. Specifically, we utilized OA-MIL (Liu et al., 2022a) as the teacher model, trained on the noisy dataset. Once the training of the teacher model was completed, we froze its parameters and employed it to perform inference on the training set, thereby generating relatively corrected labels. These corrected labels were then utilized to train the student model. The student model shared the same structure as the teacher model but benefited from the more accurate corrected labels, enabling further refinement of the bounding box positions.

**Figure 6:**
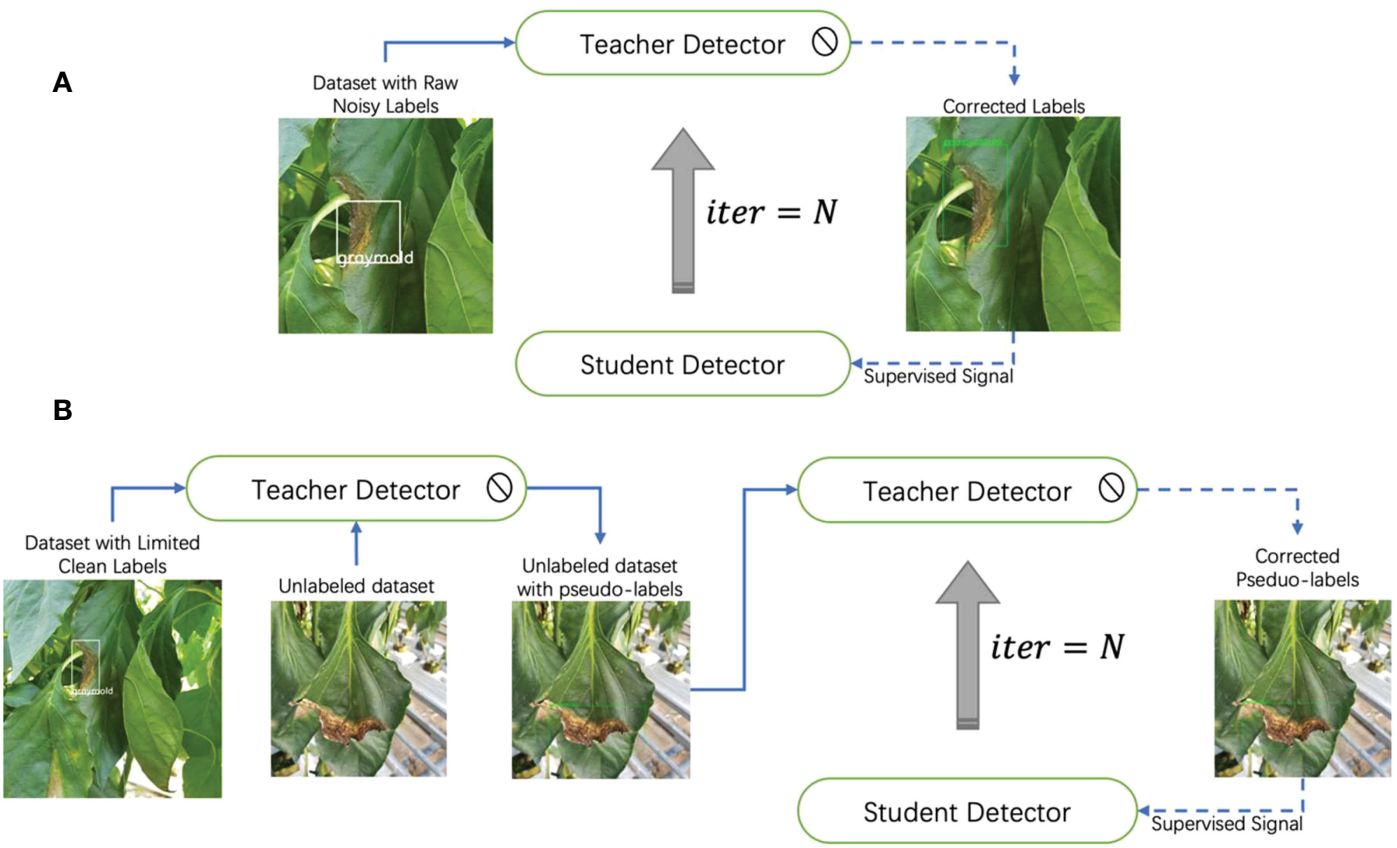
Iterative teacher-student learning framework. **(A)** The teacher detector corrects the noisy annotation. The corrected annotation is used as a supervised signal to train a student detector. Once the student detector is well-trained, it will become a new teacher. **(B)** The pipeline for extending our method to semi-supervised learning tasks. We use OA-MIL as teacher and student detector.

Once the student model was well-trained with the correct annotations, it served as the teacher detector to further refine the annotations. This iterative process of teacher-student learning and annotation refinement helped improve the accuracy and reliability of the final annotations. This process is repeated N times. We have observed that increasing the number of iterations does not lead to a sustained improvement in model performance. In our experiments, we found that the label set typically reached its peak performance after three iterations. Beyond this point, the model’s performance tended to plateau, indicating diminishing returns in terms of performance gains with further iterations. In this paper, the default setting for N was three. The corrected annotations were used as a supervised signal for the student detector, which improved the model’s performance by a continuously optimized label set. The localization performance of the student detector was thus improved. Compared to Faster-RCNN, our model does not incur any additional computation during the inference phase, resulting in no extra latency. The overall framework is shown in **Figure 6**.

### 2.5 Extension to semi-supervised learning

Semi-supervised learning aims to train object detectors with a large scale of image-level annotations and some box-level annotations (Liu et al., 2022a) (Li et al., 2022). Generally, there are mainly three steps in the semi-supervised learning paradigm. Firstly, training the model on limited labeled data; then, pseudo-labels are generated for the unlabeled data by using the output of the previous step; finally, optimizing the quality of the pseudo-labels to perform semi-supervised learning tasks. We argue that pseudo-labels can be treated as inaccurate instance-level annotations. In other words, we transform the optimization problem of pseudo-labels into a label correction problem. Therefore, extending our method naturally to the semi-supervised learning paradigm is meaningful and feasible. However, the model’s performance is limited by annotated data scale, thereby sufficient mislabeled or wrongly localized box predictions are selected as pseudo-ground truth, resulting in a sub-optimal solution of detection performance. As shown in **Figure A.2**, the model may generate predictions of two classes for one symptom.

We observed that without cleaning up redundant labels during the iterative process, it increased the number of false positive boxes in subsequent iterations. To overcome this issue, we implemented a post-processing step for the pseudo-labels using prior knowledge. We iterated through all the predicted pseudo-labels and set an intersection-over-union (IoU) threshold 0.3. For a pair of predicted bounding boxes with different categories, we retained only the one with the higher confidence score as the pseudo-label for the next iteration, discarding the lower-scoring one. While for a pair of predicted bounding boxes with the same category, we retained the big one. This post-processing step helped refine the pseudo-labels and improve the overall quality of the iterative process. The second row in **Figure A.2** showcases the pseudo-labels retained after applying the label post-processing method.

Additionally, the model paid special attention to some samples’ healthy, unknown, and background regions, which is exacerbated when the available labels are limited. As shown in **Figure A.3**, the model generated many false positive bounding boxes in these regions. To address this issue, we introduced additional control class labels such as background class, unknown class, and healthy label. These control class labels were added to improve the model’s decision-making capability and mitigate the generation of false positive predictions.

During the data annotation process, we can initially annotate a subset (e.g. 1%) and then use our proposed method to infer and save pseudo-labels. The process of preserving pseudo-labels is commonly referred to as auto-labeling (Zhang et al., 2021). Through auto-labeling, researchers can obtain a large number of pseudo-labels. These pseudo-labels have relatively high quality and require minimal further data cleaning to obtain high-quality labels. Auto-labeling enables researchers to efficiently annotate data, accelerating the annotation process and facilitating the development of models with improved performance.

### 2.6 Evaluation metric

Intersection-over-Union metric (IoU): We utilized a threshold of 0.5 to capture true positive detections generated by the model, as:


(6) $$\text{IoU}=\left|\frac{\text{A}{\;}^{\cap }\text{B}}{\text{A}{\;}^{\cup }\text{B}}\right|$$

where A and B represent the ground truth and predicted box, respectively.

Mean Average Precision score (mAP): mAP is the area under the precision-recall curve calculated for all classes. We utilize the standard mean average precision (mAP) metric with an intersection over union (IoU) threshold of 0.5 (mAP@50).


(7) $$\text{AP}=\frac{1}{11}\sum_{\text{r}\in \left[0,0.1,\ldots ,0.9,1\right]}\text{P}\left(\text{r}\right)$$

(8) $$P\left(r\right)=\underset{\tilde{\text{r}}:\tilde{\text{r}}\geq \text{r}}{\text{max}}\text{ p}\left(\tilde{\text{r}}\right)$$

where, P(r) is the maximum precision for any recall values greater than r, and $p\left(\tilde{r}\right)$ is the measured precision at recall $\tilde{r}$ .

## 3 Experiments and results

### 3.1 Implementation details

#### 3.1.1 Noise analysis

We utilized the LXML library to read clean annotation and noisy annotation files. For each label pair, we computed the intersection between each clean bounding box and all noisy bounding boxes under the same category by using IOU function. Then we selected the largest intersection as the matching box.

#### 3.1.2 Detector

We benchmarked Faster-RCNN (Ren et al., 2015), a representative two-stage detector. All experiments are based on the popular open-source code libraries mmdetection (Chen et al., 2019) and OA-MIL (Liu et al., 2022a) with default settings. A distributed training method was adopted with a batch size of 8 per GPU 3090. The learning rate was set to 0.02. ResNet-50 (He et al., 2016) is used as a default backbone and initialized with weights pre-trained on ImageNet.

#### 3.1.3 Annotation correction

The model was trained for 12 epochs in one iteration, and the learning rate was reduced by a factor of 0.1 at the 8th and 11th epochs. In the experiments regarding label correction, no offline data augmentation was employed. Instead, only horizontal image flipping was employed as online training data augmentation. We estimate the time complexity of the model using training duration. Over 4742 training images, our model requires only 1.42 hours to complete training, approximately 1.2 times faster than Faster-RCNN (1.17 hours). The overall time complexity depends on the number of iterations, and with the number of iterations set to 3, the training duration is approximately four times that of Faster-RCNN (including label correction and updates).

#### 3.1.4 Semi-supervised learning

When 1% labeled data was available, the model was trained for 200 epochs, and the learning rate was decreased by 0.1 at the 150th and 180th epochs. When 10% labeled data was available, the model was trained for 100 epochs, and the learning rate was decreased by 0.1 at the 75th and 90th epochs. Horizontal image flipping, vertical flipping, 90-degree rotation, and random scaling were used as offline training data augmentation for labeled data. Following the completion of the initial training, the model was utilized to infer over all unannotated images within the training set. We used the LXML library to save pseudo-label files for automatic annotation. Subsequent iterations were conducted using the default settings for label correction. While this approach may require three times the training duration compared to Faster-RCNN, it offers significant cost savings regarding manual annotation.

#### 3.1.5 Inference

Only the final student detector was needed in the inference stage, so there was no additional computational cost. This indicates that our model has the same inference speed as the baseline model Faster R-CNN. All configurations at this stage can be performed according to the default settings without additional modification. The IoU threshold for non-maximum suppression was set to 0.5, and the score threshold was 0.5 which is higher than the default setting of 0.05 in most works.

### 3.2 Annotation correction results

For a fair comparison, all models were evaluated on the well-annotated paprika disease test set in (Dong et al., 2022). The root mean square relative error γ was set to 0.1, 0.15, and 0.2, respectively, to generate datasets with synthesized noise and evaluate the method’s effectiveness. The performance of Faster-RCNN in various settings was used as a benchmark. **Table 2** shows the result on real-world noise datasets and synthesized noisy datasets. Note that we performed validation experiments on synthetic noise. The results presented in **Table 2** are the mean of two experiments. Surprisingly, our method achieved a slight improvement even on clean datasets.

**Table 2:** Performance comparison on the paprika disease test dataset. γ denotes the synthesized noise level.

Methods (mAP@50(%))	Noise Type				
	Clean (*γ* = 0)	Real-world (*γ* ≈ 0.15)	Synthesized (*γ* = 0.1)	Synthesized (*γ* = 0.15)	Synthesized (*γ* = 0.2)
Faster-RCNN (Ren et al., 2015)	90.2	57.1	75.6	58.5	48.2
YOLO-v8-L (Jocher et al., 2023)	**91.0**	67.2	79.6	66.8	53.9
NDet (Wang et al., 2022b)	90.6	78.7	81.4	78.2	73.8
OA-MIL (Liu et al., 2022a)	90.6 (+0.4)	77.8 (+20.7)	80.2 (+4.6)	76.5 (+18.0)	71.6 (+23.4)
Ours	90.6 (+0.4)	**83.1 (+26.0)**	**84.3 (+8.7)**	**82.1 (+23.6)**	**78.4 (+30.2)**

Parentheses indicate the performance improvement of our method compared to the Faster RCNN. The best performance is in boldface. We use mAP@50(%) as performance metric to evaluate our model.

Our method can enhance the model’s robustness to location noise. Under real-world noise, the performance improved from 57.1% to 83.1%. With synthetic noise at γ=0.15 , our model improved from 58.5% to 82.1%, which is almost the same gain trend as real-world noise. Therefore, it is reasonable to describe the noise level by the root mean square relative error γ . Compared with OA-MIL, our model improved by 5.3%. It indicated that inaccurate ground truth dominates strong object localization priors, thereby misleading the correction module in OA-MIL. Thus, optimizing a model by itself by only learning corrected labels cannot achieve optimal performance since corrected labels still contain noise. Our method used a teacher-student learning framework to correct the noisy labels and finally trained a more robust model. Extensive experiments verify the effectiveness of our method. To benchmark against state-of-the-art detectors, we provide the performance on YOLOv8-L (Jocher et al., 2023), comparable in parameter count to Faster R-CNN. Additionally, we compare our approach with the noise correction method NDet (Wang et al., 2022b) based on Faster R-CNN. The results indicate that these methods may perform better on clean datasets or datasets with lower noise levels. However, our approach demonstrates a clear advantage as the γ increases.

**Figure 7:**
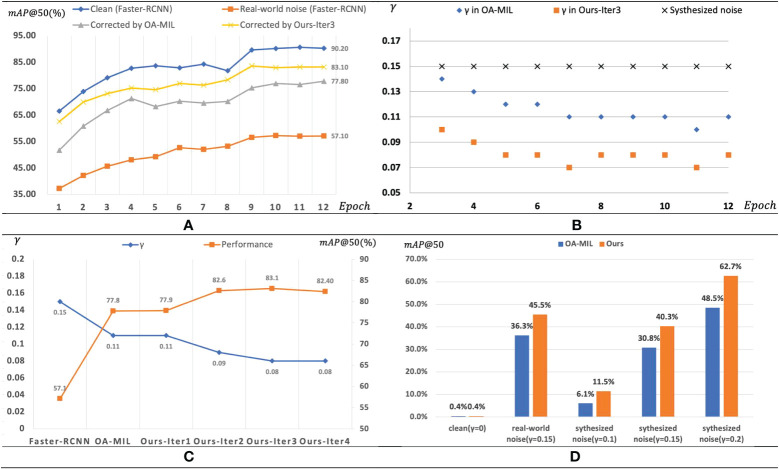
Training process. **(A)** mAP evaluated on paprika disease test set at each epoch for different training scenarios. **(B)** The root mean square relative error *γ* of training samples in these training scenarios. We assume *γ* equals 0 in the clean dataset. **(C)** Comparison of our method with OA-MIL. **(D)** Comparison of performance change rates of OA-MIL and Ours over Faster-RCNN.

As shown in **Figure 7**, the performance according to the number of training epochs and the number of iterations was compared using a clean and real-world noisy paprika dataset. Faster-RCNN was used as the baseline detector. **Figure 7A** shows the mAP comparison of different datasets used for training according to the number of training epochs. Performance increases as the epoch increases in all datasets. Using clean datasets for training shows the best performance, and using real noisy datasets shows the worst performance. The proposed method shows better performance of the two types of label correction methods. The graph for the proposed method with an iterative process shows the results in the training period of the student detector in the last iterative process. **Figure 7B** shows the change of γ over epochs. Although noisy bounding boxes corrected by OA-MIL (Liu et al., 2022a) have a lower γ than the noisy annotations, only relying on the annotations corrected to provide appropriate supervision still cannot achieve the optimal performance of the model. **Figure 7C** shows how γ and mAP change with iterations of the proposed method. Our method can further correct noisy labels, thereby iteratively improving model performance. We can see that γ decreases and mAP increases as the iteration proceeds to some extent, which shows that the proposed iteration method is effective.

Besides, **Figure 7D** shows the comparison of performance change rates of OA-MIL (Liu et al., 2022a) and ours over Faster-RCNN. Note that our method achieved more remarkable performance gain at higher noise levels, which undoubtedly alleviated the adverse effects of location noise, thereby narrowing the performance gap caused by noise. We can even conclude that if the performance of Faster-RCNN differs greatly from our method, the dataset may contain severe noise interference.

### 3.3 Semi-supervised learning results

The pseudo-labels generated during semi-supervised learning also contain localization noise. Therefore, our method can be easily generalized to the semi-supervised learning paradigm naturally. We randomly chose 1%, 10%, and 50% of the clean training label set as available labeled data and the remaining data as unlabeled data. **Table 3** shows the corresponding experimental results. For the paprika dataset, our method achieved approximately 75% of the performance of a fully supervised object detector when 1% of labels are available. In comparison, with 10% available labels, our method achieved 86% of the performance equivalent to fully supervised object detectors.

**Table 3:** The result of semi-supervised learning scenario setting on different datasets. The last row is the ratio of ours to Faster-RCNN*.

	Paprika disease (mAP@50) (%)			GWHD2021 (mAP@50) (%)		
Faster-RCNN*	90.2			56.0		
Labeled data	1%	10%	50%	1%	10%	50%
Faster-RCNN	54.6	69.9	75.2	33.6	46.0	48.2
Ours	67.5	77.6	82.9	42.1	50.5	53.4
(Efficiency)	(74.8%)	(86.0%)	(91.9%)	(75.2%)	(90.2%)	(95.3%)

* denotes the result of training Faster-RCNN on the full (100%) clean dataset.

Furthermore, despite our post-processing techniques and adding control categories, we observed that classification errors persist in the pseudo-labels generated by this method. We attribute this to the limited data volume and multi-class classification problem. In other words, our method pays more attention to annotation localization noise rather than class noise. The GWHD2021 dataset only contains annotations for wheat heads and no other categories. For rigor, the GWHD2021 dataset was also used to validate the method’s performance in a semi-supervised learning setting. Our method achieved 90.2% of the performance of a fully supervised object detector when 10% of the labels are available, reflecting our method’s efficiency. Therefore, the annotation cost can be significantly reduced by deploying our method on semi-supervised learning tasks.

### 3.4 Ablation study

This section explores the impact of different components or design choices in our approach. Noise-corrected ablation experiments are based on Faster-RCNN with synthetic Gaussian noise ( γ=0.15 ). As mentioned in Section 2.4, relying only on the predictions produced by the detector itself as a source of supervision may not lead to optimal solutions. Therefore, we propose a teacher-student learning framework. **Table 4** shows the number of iterative trainings versus model performance. We observed that too many iterations could lead to model performance degradation. Therefore, we iterated three times to stop for the final result. Unless otherwise specified, all experimental results in the paper are reported based on the results obtained after three iterations.

**Table 4:** The choice of the number of iterations for teacher-student learning trained on the paprika disease dataset. γ denotes the synthesized noise level.

Methods	Clean	Real-world noise	Synthesized noise		
	γ=0	γ≈0.15	γ=0.1	γ=0.15	γ=0.2
Faster-RCNN	90.2	57.1	75.6	58.5	48.2
Ours(iter-1)	90.6	77.9	79.2	76.5	71.6
Ours(iter-2)	–	82.6	84.1	81.6	78.5
Ours(iter-3)	–	**83.1**	**84.3**	**82.1**	**78.4**
Ours(iter-4)	–	82.4	83.5	81.6	77.9
Ours(iter-5)	–	82.7	83.2	81.5	77.5

We used mAP50 to validate the performance at different iteration times. The best performance is in boldface.

In semi-supervised learning tasks, we added control class labels, pseudo-label post-processing, and data augmentation to the noisy dataset to improve the discriminative power of the model. It is essential to adopt the post-label processing process for the iterative training of teachers and students in the later stage. Otherwise, the model will generate many overlapping or wrong labels and cause the task to fail. **Table 5** shows the result of ablation experiments on semi-supervised learning. We have presented the qualitative results of model post-processing and class control in **Figures A.2**, **A.3** in the appendix.

**Table 5:** Ablation studies in a semi-supervised learning setting.

Faster-RCNN	Data Augmentation*	Post-process	Control Class	Teacher-student	Paprika Disease mAP@50(%)	
					1%	10%
√	√				54.6	69.9
√	√			iter-1	55.8	61.0
√	√	√		iter-1	56.1	61.5
√	√	√	√	iter-1	57.3	64.2
√	√	√	√	iter-2	66.9	**78.4**
√	√	√	√	iter-3	**67.5**	77.6
√	√	√	√	iter-4	67.4	77.9
√	√	√	√	iter-5	67.1	77.4

The models are trained on the paprika disease clean dataset. The best performance is in boldface. The * denotes only performing offline data augmentation on the labeled set.

### 3.5 Visualization

The distributions of relative boundary coordinate errors for noisy annotations and our corrected ones are shown in **Figure 2**. The relative error of noisy annotations became smaller after being corrected by our method. Besides, we also visualize the experimental results in Appendix. In **Figure A.1**, each triplet contains input annotations, OA-MIL corrected results, and our method corrected results. The annotation correction results of our method cover the actual object more tightly than OA-MIL’s. **Figure A.2** demonstrates that post-processing of pseudo-label can remove low-confidence annotations and overlapping labels. **Figure A.3** presents that the model reduces the misjudgment of suspicious regions after adding the control category. **Figure A.4** displays the pseudo-labels generated by our method in the semi-supervised setting, where the last two rows show some failed predictions. The model is more prone to misclassify instances with similar symptoms, which may be due to the limited dataset. Even with misclassifications, the labels still closely match the actual objects, demonstrating the robustness of our method to locations. **Figure A.5** shows the pseudo-labels generated by our method on the unlabeled dataset in the wheat head classification dataset GWHD2021. Our method can locate most of the wheat heads accurately.

## 4 Discussion

### 4.1 Does human cost equal intelligence?

In computer vision, there is an old saying highlighting the importance of labeling in deep learning methods: “As much human cost, there is as much machine intelligence.” Labeled datasets have played a crucial role in the rapid development of deep learning. Over the years, researchers have proposed methods to reduce computation costs and improve intelligence, such as novel feature extractors, optimized loss functions, and efficient augmentation strategies. These methods have significantly contributed to the advancement of related industries.

**Figure 8:**
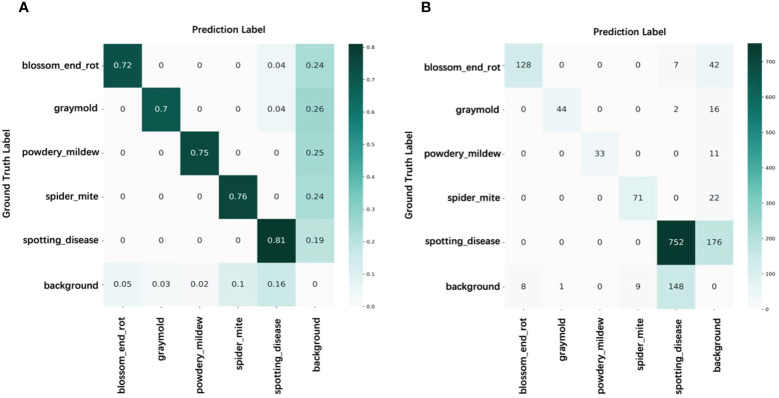
Confusion matrix of pseudo-label on paprika disease training set with 1% labeled data. **(A)** Percentage count results. **(B)** Count results.

Nevertheless, in specific domains, inaccuracies in annotations pose substantial hurdles for these methodologies. Our approach tackles this challenge by embracing a semi-supervised learning framework, which facilitates automatic annotation and fortifies the model against localization errors. This dual benefit notably slashes the costs associated with manual annotation. **Figure 8** depicts the confusion matrix of the pseudo-labels for unlabeled samples when only 1% of the training set is labeled. The results show that backgrounds and some unseen instances are often misclassified as known classes, while some instances are overlooked due to limited diversity in the labeled data. Regarding automatic labeling, further corrections for false positives and negatives are required to achieve comparable performance to fully supervised learning methods. Manual annotation is unnecessary for well-learned instances as it would be a waste of resources.

### 4.2 Limitations and future work

In our previous studies (Dong et al., 2022), we established that localization noise exerts a more pronounced impact on model performance than class noise. Therefore, the core objective of this research was to address the challenge of correcting inaccurate bounding box annotations caused by localization noise. It is important to note that while our emphasis is on localization noise, it does not suggest the absence of class noise within real-world label sets. In practice, both types of noise can coexist, posing significant hurdles in training accurate models for object detection tasks. More research should focus on the patterns of real-world noise, based on which more effective methods can be proposed and further improve the detector performance. However, quantifying class noise is very difficult due to the diversity of datasets. Meanwhile, adding class noise in a random perturbation way does not match the actual distribution of class noise. Therefore, before the methodology is proposed, how to construct a class noise dataset is a question worth considering.

Recently, large-scale vision-language models (Zhang et al., 2022; Kim et al., 2023) have been applied to localization tasks. These models can locate objects in images using only textual labels, without the need for explicit training. This process is known as zero-shot inference (Cao et al., 2023). In the semi-supervised setting of this paper, we require pre-labeling a portion of the data to train the teacher model for automatic annotation. In contrast, it may be possible to achieve fully automatic annotation by correcting the zero-shot inference results of these large-scale vision-language models. Therefore, correcting the zero-shot inference results of these models is an interesting research topic that we plan to explore in future work.

## 5 Conclusion

This paper investigated the impact of location noise on detector performance in real-world environments. We observed that relative location noise in real scenarios follows Gaussian distribution and is dependent on object size, which guides how to synthesize location noise. Furthermore, we proposed an annotation correction method based on the teacher-student learning paradigm, which significantly narrows the performance gap caused by noise. Utilizing our method is crucial for performance improvement if the labeling budget is limited or constrained. Our method also supports the correction of imprecise pseudo-labels generated in a semi-supervised learning task, implying that our method can be extended to semi-supervised learning tasks. In summary, our method is suitable for handling datasets with low-quality annotations, thus reducing the annotation cost and improving traditional labeling methods.

## Data availability statement

The raw data supporting the conclusions of this article will be made available by the authors, without undue reservation.

## Author contributions

JD designed the method, performed the experiments, and wrote the manuscript. AF and DP provided support in the data collection, plant disease knowledge, and proofreading article. SY and HK advised in the design of the system and analyzed the annotation strategies to find the best method for efficient plant disease detection. All authors contributed to the article and approved the submitted version.

## Supplementary material

Supplementary Figure 1 Comparison of our method with OA-MIL results. The first row displays labels with location noise. The second and third rows show the correction results using OAMIL and our method (iter-3). The labels in the third row are more closely aligned with the actual disease locations.

Supplementary Figure 2 Example results of post-processing the pseudo-labels. We use red color to highlight false positive bounding boxes. Better displayed on the screen.

Supplementary Figure 3 The model reduces the number of false positive labels for suspicious regions by adding control classes. When the data volume is low, the model is prone to classifying backgrounds, unknown regions, and healthy areas as diseases. The red color highlights false positive bounding boxes. Better displayed on the screen.

Supplementary Figure 4 Pseudo-labels generated in a semi-supervised learning setting when only 1% of the label set is available on the paprika disease dataset. The last two lines present some failure cases, including classification failures, localization failures, and missed detections.

Supplementary Figure 5 Pseudo-labels (after three iterations) generated in a semi-supervised learning setting when only 1% of the label set is available on the GWHD dataset.
