# Supplementary material for: An iterative noisy annotation correction model for robust plant disease detection
Source: Front Plant Sci. 2023 Oct 13;14:1238722. doi: 10.3389/fpls.2023.1238722 (PMC10628849; doi:10.3389/fpls.2023.1238722)
Supplement: Supplementary Figure 1 — Comparison of our method with OA-MIL results. The first row displays labels with location noise. The second and third rows show the correction results using OAMIL and our method (iter-3). The labels in the third row are more closely aligned with the actual disease locations. [file Presentation_1.zip › supplementary material_v2.docx]

**Appendix:**

**
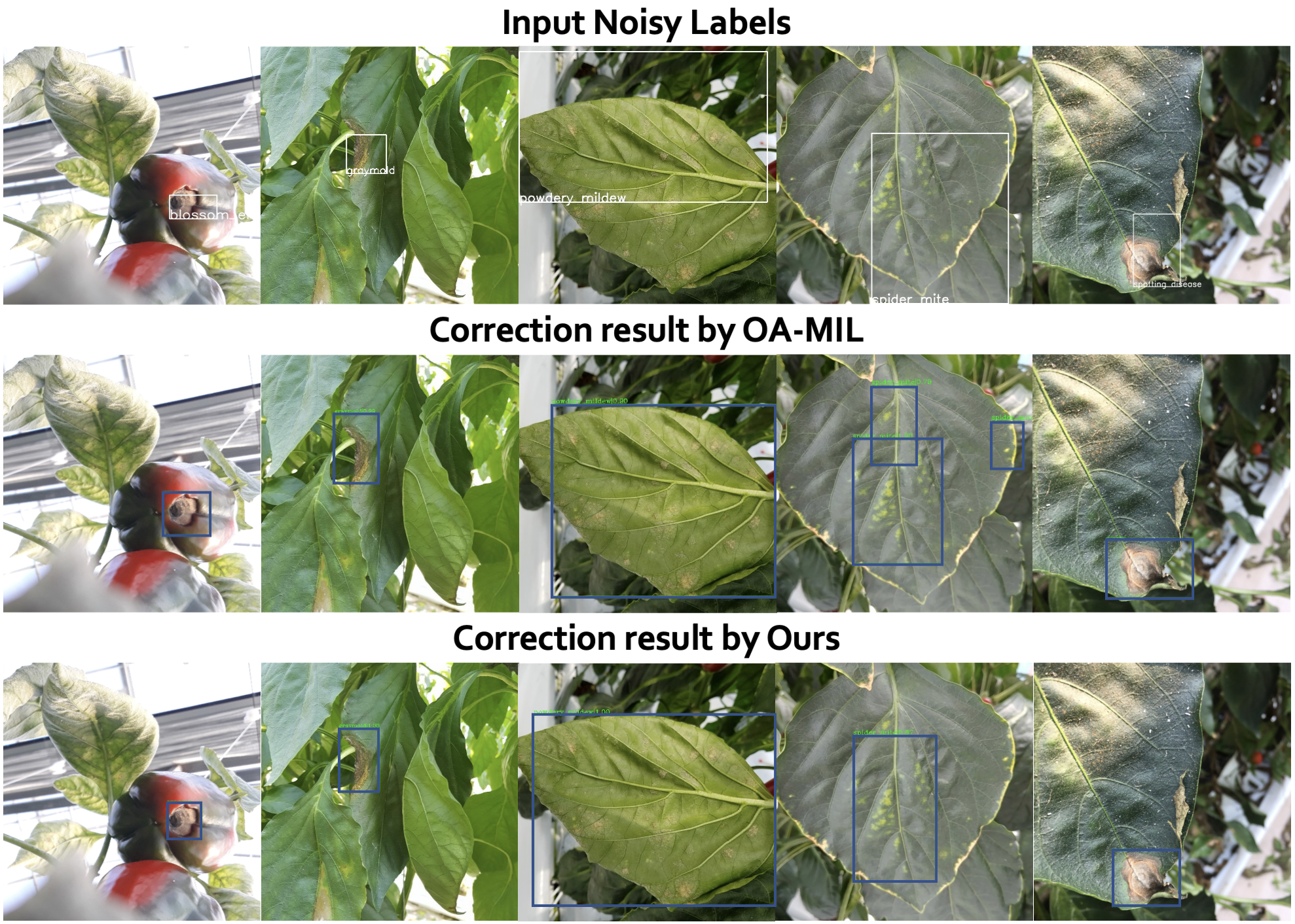
**

**Figure A.1.** Comparison of our method with OA-MIL results. The first row displays labels with location noise. The second and third rows show the correction results using OAMIL and our method (iter-3). The labels in the third row are more closely aligned with the actual disease locations.

**
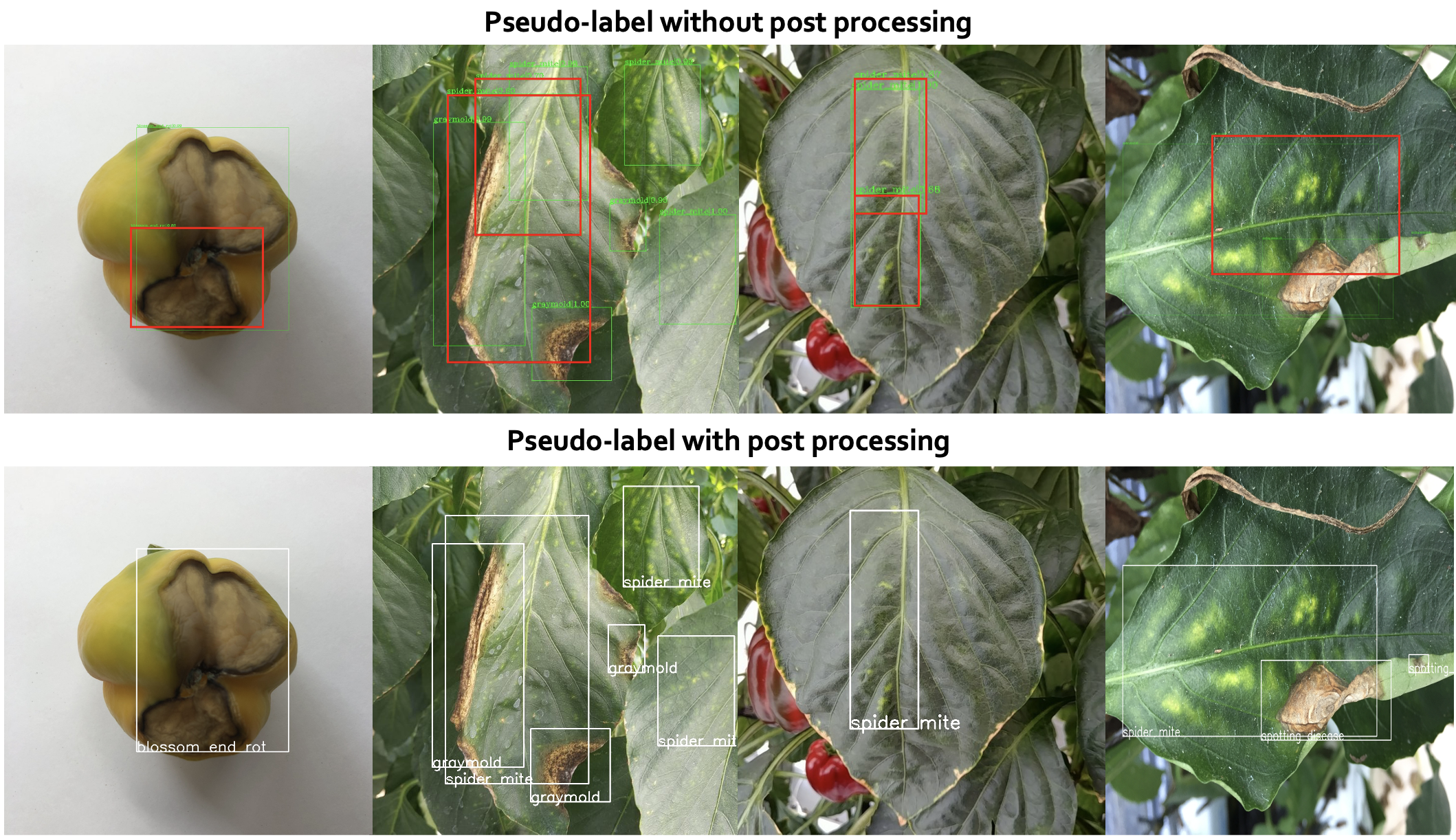
**

**Figure A.2.** Example results of post-processing the pseudo-labels. We use red color to highlight false positive bounding boxes. Better displayed on the screen.

**
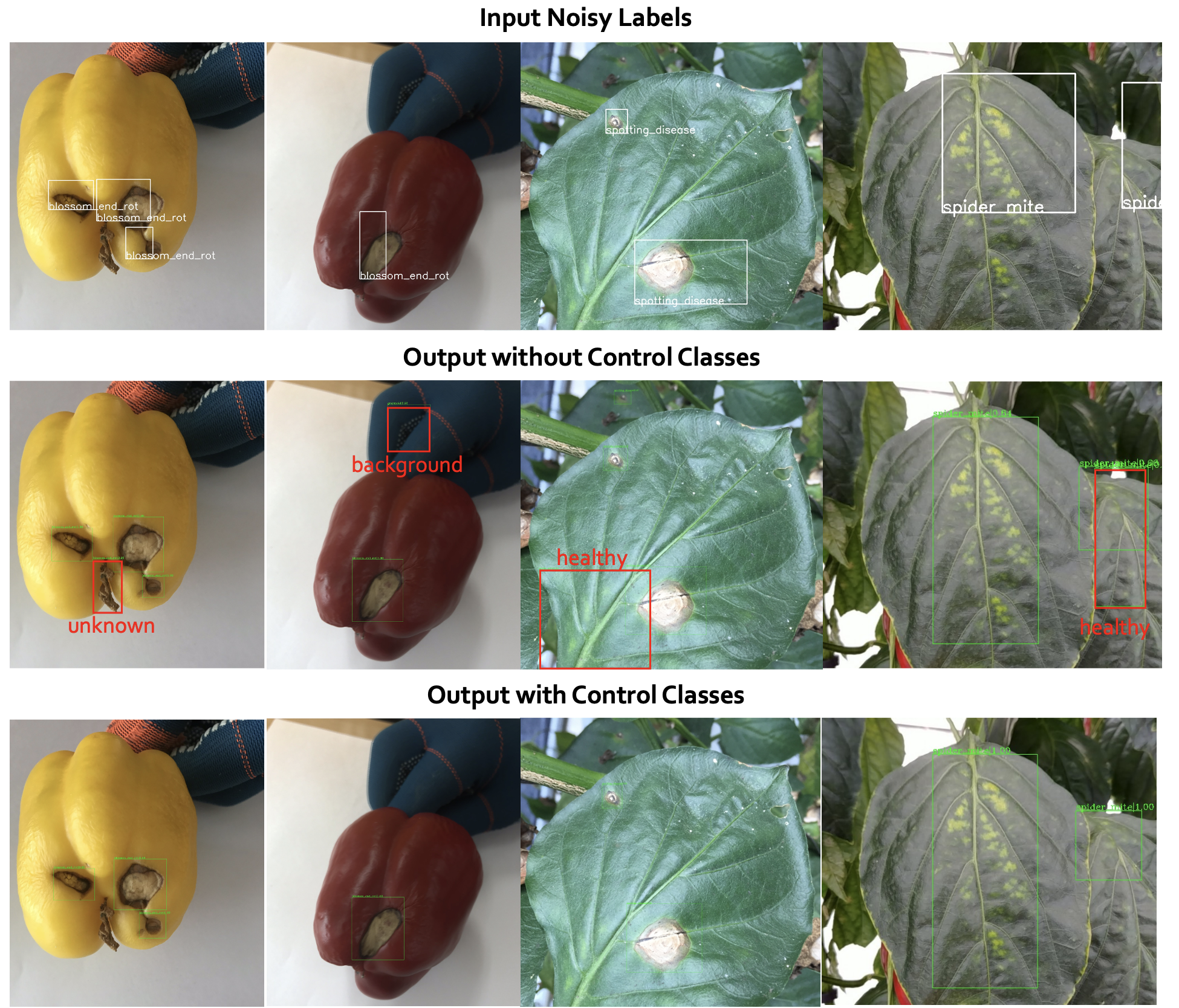
**

**Figure A.3.** The model reduces the number of false positive labels for suspicious regions by adding control classes. When the data volume is low, the model is prone to classifying backgrounds, unknown regions, and healthy areas as diseases. The red color highlights false positive bounding boxes. Better displayed on the screen.

**
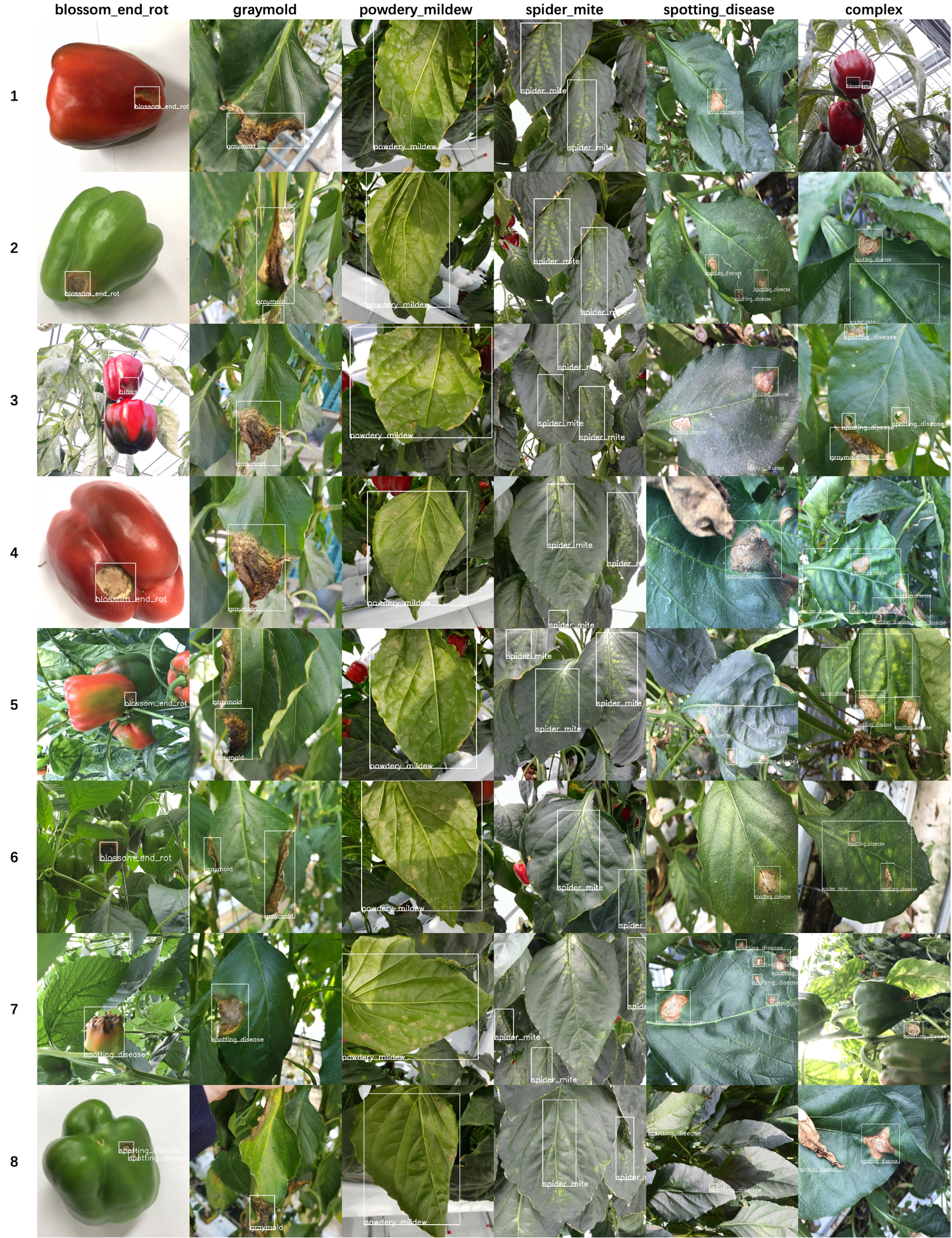
**

**Figure A.4.** Pseudo-labels generated in a semi-supervised learning setting when only 1% of the label set is available on the paprika disease dataset. The last two lines present some failure cases, including classification failures, localization failures, and missed detections.

**
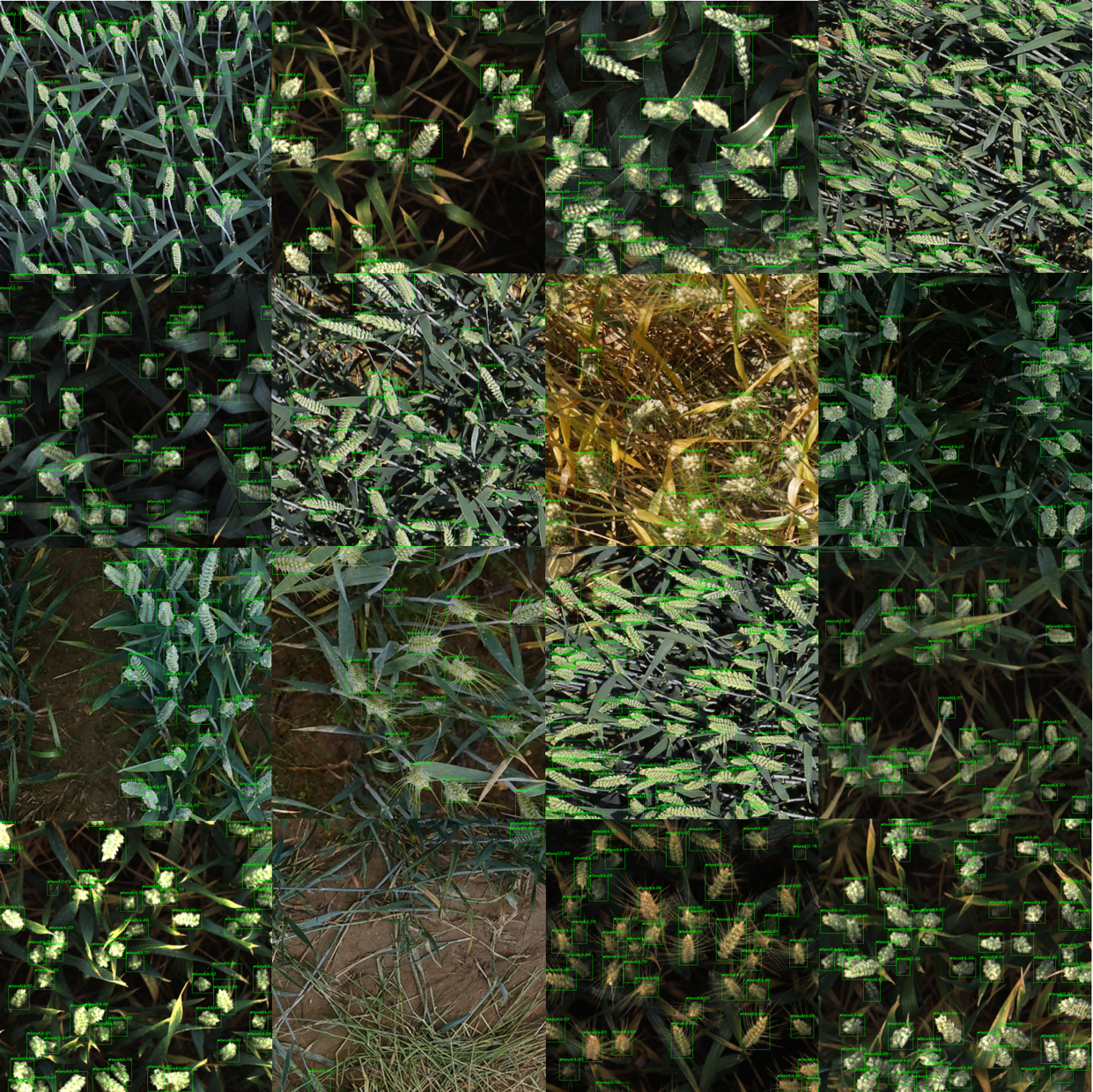
**

**Figure A.5.** Pseudo-labels (after three iterations) generated in a semi-supervised learning setting when only 1% of the label set is available on the GWHD dataset.
